# Pancreatic cancer risk in relation to sex, lifestyle factors, and pre-diagnostic anthropometry in the Malmö Diet and Cancer Study

**DOI:** 10.1186/s13293-016-0120-8

**Published:** 2016-12-09

**Authors:** Gustav Andersson, Christoffer Wennersten, Signe Borgquist, Karin Jirström

**Affiliations:** Division of Oncology and Pathology, Department of Clinical Sciences, Lund, Lund University, Skåne University Hospital, 221 85 Lund, Sweden

**Keywords:** Obesity, Alcohol, Smoking, Lifestyle, Pancreatic cancer risk

## Abstract

**Background:**

Lifestyle factors may influence the risk of developing pancreatic cancer. Whereas cigarette smoking is an established risk factor, the effects of high alcohol intake and obesity are more uncertain. The aim of the present study was to examine the associations of pre-diagnostic anthropometry, alcohol consumption, and smoking habits with pancreatic cancer risk in a Swedish prospective, population-based cohort, with particular reference to potential sex differences.

**Methods:**

The studied cohort consists of 28,098 participants, including all incident cases of pancreatic cancer, in the Malmö Diet and Cancer Study up until December 31, 2013 (*n* = 163). Non-parametric and chi-squared tests were applied to compare the distribution of risk factors between cases and non-cases. Cox regression proportional hazards models were used to estimate the relationship between investigative factors and pancreatic cancer risk. Anthropometric factors included height, weight, body mass index (BMI), waist and hip circumference, waist-hip ratio (WHR), and body fat percentage.

**Results:**

BMI was not a significant risk factor for pancreatic cancer, but a higher WHR was significantly associated with an increased risk in the entire cohort (hazard ratio (HR) 2.36, 95% confidence interval (CI) 1.28–4.35, *p* for trend = 0.009). Regular smoking was a significant risk factor among both women (HR 2.62, 95% CI 1.61–4.27) and men (HR 3.57, 95% CI 1.70–7.47), whereas occasional smoking was a significant risk factor only in women (HR 3.29, 95% CI 1.50–7.19). Passive smoking at work for >20 years was significantly associated with an increased risk in the entire cohort (HR 1.73, 95% CI 1.15–2.58) and in women selectively (HR 2.01, 95% CI 1.21–3.31). Alcohol consumption was not a significant risk factor. A significant interaction was found between female sex and age (*p* = 0.045), but no other factor, in relation to pancreatic cancer risk.

**Conclusions:**

WHR was the only pre-diagnostic anthropometric factor associated with pancreatic cancer risk, with no sex-related differences. Regular smoking was confirmed as a significant risk factor in both sexes, whereas occasional and passive smoking were significant risk factors only in women. Despite the lack of a significant interaction between smoking and sex in relation with pancreatic cancer risk, potential sex differences should be considered in future epidemiological studies.

## Background

In 2012, pancreatic adenocarcinoma represented 3% of all cancer cases in developed countries, non-melanoma skin cancer excluded [1]. Despite this, it was the fourth leading cause of cancer-related death, and responsible for 330,400 deaths worldwide [1]. This demonstrates the poor prognosis of the disease, with a 5-year overall survival (OS) of only 4%, and most patients decease less than 12 months after confirmation of the diagnosis [2]. According to the latest cancer statistics report from the National Board of Health and Welfare in Sweden (2014), the peak incidence of pancreatic cancer occurs at an age of 70–74 years, with the highest incidence rate at 75–79 years of age [3]. The incidence of pancreatic adenocarcinoma has for a long period of time been higher in men than in women [4–6], but during the last decades the numbers have become more even, and today there is no longer any evident sex-related difference [3, 5, 7].

Several risk factors for pancreatic cancer have been reported; however, only a few of them have been confirmed more consistently. Tobacco smoking seems to be the only established modifiable risk factor [7–10], but high age [2, 4, 8] and heredity for pancreatic cancer [11, 12] are also often referred to as well-established risk factors. Diabetes [13–15] and pancreatitis [7, 16, 17] are thereafter the most commonly studied, but still debated, risk factors. The possible influence of body constitution (anthropometry) on the risk of developing pancreatic cancer has also been a subject for discussion, and several studies have demonstrated a significant association between high body mass index (BMI) and risk of pancreatic cancer [5–7, 15, 16, 18, 19]. A handful of theories to explain the plausible correlation between a high BMI and increased risk of pancreatic cancer have been presented. One of them proposes that the increased insulin resistance and the elevated levels of circulating insulin in overweight patients stimulate growth of pancreatic tissue, which increases the risk of neoplasms to arise [5]. However, several studies have not been able to demonstrate a significant association between BMI and pancreatic cancer risk [20–22].

While active smoking is considered a well-established risk factor for pancreatic cancer, the risk associated with passive smoking is less clear. In a previous study based on The European Prospective Investigation into Cancer and Nutrition (EPIC) cohort [23], environmental tobacco smoking (ETS) during childhood *and* at work/at home in adult life was found to significantly increase the risk of pancreatic cancer among never smokers, compared to those never exposed. Exposure to ETS at home or at work in adult life was borderline significantly associated with pancreatic cancer risk, but if only exposed during childhood, there was no increased risk. In that study, potential differences between sexes regarding ETS were not considered.

The association between alcohol consumption and risk of pancreatic cancer is also under debate, and the hitherto published results are inconclusive. Some studies demonstrate an increased risk of pancreatic cancer in heavy alcohol drinkers [24–27], while other studies show no such association [28–30]. Since alcohol is a well-known risk factor for recurrent acute pancreatitis (RAP) [31], as well as chronic pancreatitis (CP) [32], and both RAP and CP have been validated as significant risk factors for pancreatic cancer in several studies, a link between alcohol and pancreatic cancer incidence would not be unexpected. In a large prospective study from the USA [32], where the correlation between alcohol consumption and both RAP and CP was investigated, the correlation between alcohol consumption and CP was found to be more pronounced in men than in women, which has been shown in other studies as well [33]. A number of studies have shown the correlation between high alcohol consumption and increased pancreatic cancer risk to be significant among men [25–27], but very few have found it to be significant among women [24].

The aim of the present study was to examine the associations of anthropometric factors, smoking and alcohol consumption, with pancreatic cancer risk in the Malmö Diet and Cancer Study, with particular reference to potential differences between sexes.

## Methods

### The cohort

The Malmö Diet and Cancer Study (MDCS) is a large prospective, population-based study, which is also part of The European Prospective Investigation into Cancer and Nutrition (EPIC). MDCS/EPIC is a consecutive cohort including men and women between 44 and 73 years of age at baseline, recruited between 1991 and 1996, with a total of 28,098 participants (11,063 men and 17,035 women). The cohort has been described in detail previously [34–37].

**Fig. 1:**
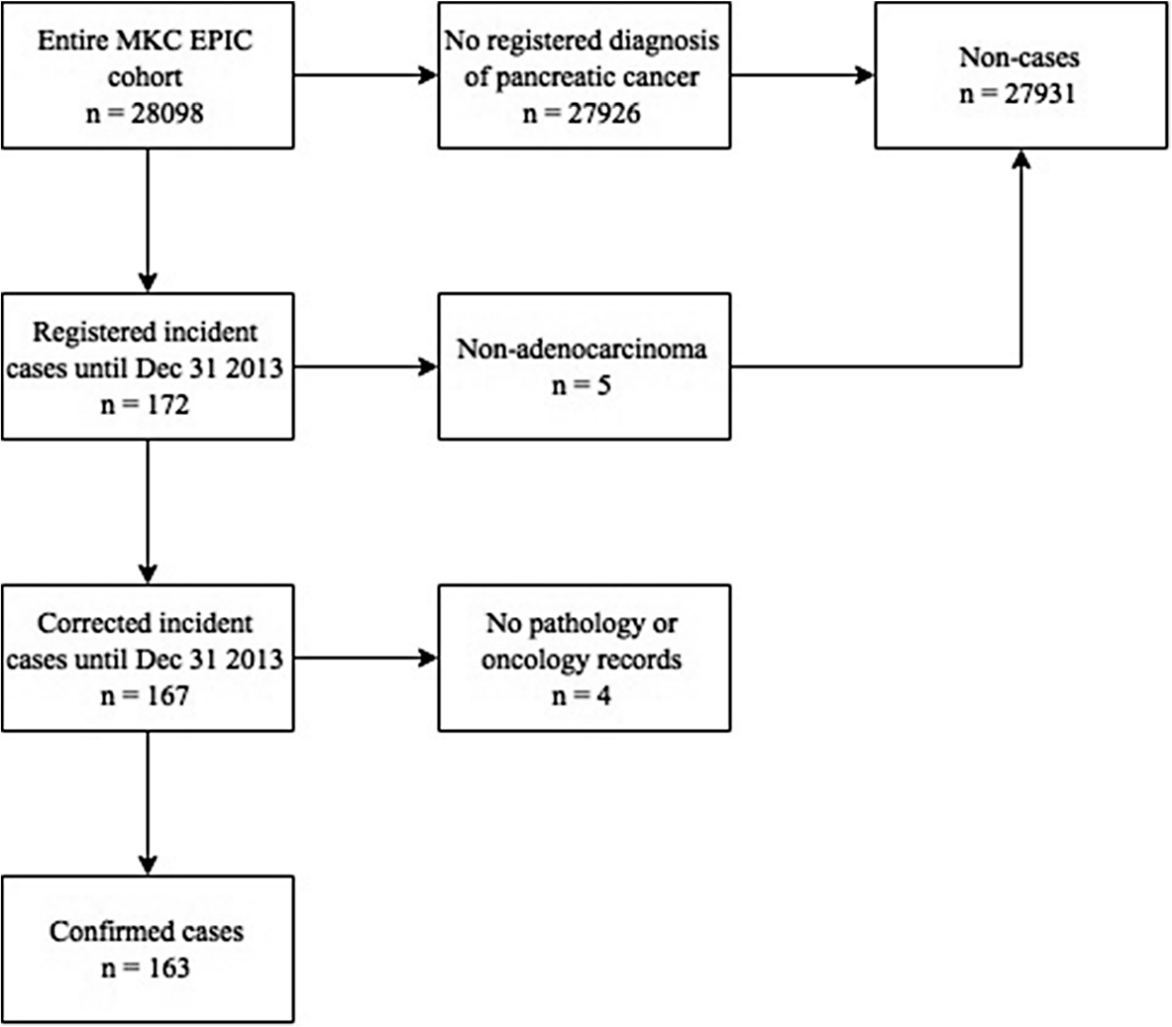
Flowchart of the cohort

From baseline until last follow-up on December 31, 2013, 172 cases of pancreatic cancer were reported from the Swedish Cancer Register among the participants. Following review of pathology records, five cases were classified as either endocrine pancreatic cancer or other types of non-adenocarcinoma and these cases were transferred to the group of non-cases. For another four cases, no pathology reports or medical records were available and the diagnosis could not be confirmed, and these were excluded from all analyses, rendering a total number of 163 cases with confirmed pancreatic cancer, 61 men (0.6% of male participants) and 102 women (0.6% of female participants). A flowchart of the cohort is shown in Fig. 1.

### Statistical methods

Cox regression proportional hazards models were applied to investigate the impact of age at baseline, sex (entire cohort), smoking habits, ETS, alcohol consumption, and diabetes with pancreatic cancer-free person-years. The analyses were performed with adjustment for age only or for all factors except ETS. The potential interaction with risk factor and sex was tested with the covariates age, sex, risk factor, and risk factor × sex, and also repeated with the fully adjusted model. Cox regression proportional hazards models were further applied to estimate the impact of anthropometric factors on pancreatic cancer-free person-years adjusted for age, sex, diabetes mellitus (DM), smoking habits, and alcohol habits. Person-years were calculated by subtracting date at baseline (date of study entry) from date at diagnosis/date at migration/date of study end (December 31, 2013).

Included anthropometric variables were height, weight, BMI, hip circumference, waist circumference, waist-hip ratio (WHR), and body fat percentage, which were all divided into tertiles for the statistical analyses. Alcohol consumption habits were recorded in grams per day, and smoking habits denoted as regularly, occasionally, former, and never. ETS was recorded as exposure during childhood (yes/no), exposure at home (no/<10 years/10–20 years/>20 years), or exposure at work (no/<10 years/10–20 years/>20 years).

In the adjusted analyses, diabetes was defined as diagnosis of diabetes more than 24 months prior to endpoint or to the date of pancreatic cancer diagnosis, separating these participants from the group of participants with new-onset diabetes, i.e., cases with confirmed diabetes less than 24 months prior to the endpoint or to the date of pancreatic cancer diagnosis.

All statistical tests were two sided. *p* values <0.05 were considered significant.

All statistical analyses were performed using IBM SPSS Statistics version 22.0 (SPSS Inc., Chicago, IL, USA).

## Results

### Baseline characteristics

The distribution of investigative factors for individuals who developed pancreatic cancer during follow-up (cases) and for those who did not (rest of cohort) is shown in Table 1.

**Table 1:** Summary of the distribution of risk factors among cases and non-cases

	Entire cohort (*n* = 28,094)		Men (*n* = 11,063)		Women (*n* = 17,031)	
Characteristics	Rest of cohort	PC cases	Rest of cohort	PC cases	Rest of cohort	PC cases
*n* (%)	27,931 (99.4)	163 (0.6)	11,002 (99.4)	61 (0.6)	16,929 (99.4)	102 (0.6)
Sex (%)						
Men	11,002 (39.4)	61 (37.4)				
Women	16,929 (60.6)	102 (62.6)				
Age at baseline (years)						
Mean	58.1	60.0	59.2	59.3	57.4	60.5
Median	57.8	59.9	59.1	58.6	56.7	61.1
Smoking (%)						
Regularly	6614 (23.7)	60 (36.8)	2602 (23.7)	25 (41.0)	4012 (23.7)	35 (34.3)
Occasionally	1250 (4.5)	11 (6.7)	529 (4.8)	3 (4.9)	721 (4.3)	8 (7.8)
Former smoker	9456 (33.9)	49 (30.1)	4758 (43.2)	23 (37.7)	4698 (27.8)	26 (25.5)
Never smoker	10,599 (37.9)	43 (26.4)	3107 (28.2)	10 (16.4)	7492 (44.3)	33 (32.4)
Missing	12 (0.0)	0 (0.0)	6 (0.1)	0 (0.0)	6 (0.0)	0 (0.0)
Environmental smoking at work						
Never	12,171 (43.6)	54 (33.1)	3746 (34.0)	14 (23.0)	8425 (49.8)	40 (39.2)
For <10 years	3277 (11.7)	20 (12.3)	1085 (9.9)	8 (13.1)	2192 (12.9)	12 (11.8)
For 10–20 years	3351 (12.0)	20 (12.3)	1251 (11.4)	8 (13.1)	2100 (12.4)	12 (11.8)
For >20 years	6208 (22.2)	51 (31.3)	3720 (33.8)	25 (41.0)	2488 (14.7)	26 (25.5)
Missing	2924 (10.5)	18 (11.0)	1200 (10.9)	6 (9.8)	1724 (10.2)	12 (11.8)
Environmental smoking at home						
Never	13,899 (49.8)	74 (45.4)	6027 (54.8)	31 (50.8)	7872 (46.5)	43 (42.2)
For <10 years	2821 (10.1)	11 (6.7)	974 (8.9)	4 (6.6)	1847 (10.9)	7 (6.9)
For 10–20 years	2973 (10.6)	20 (12.3)	959 (8.7)	9 (14.8)	2014 (11.9)	11 (10.8)
For >20 years	5242 (18.8)	40 (24.5)	1828 (16.6)	11 (18.0)	3414 (20.2)	29 (28.4)
Missing	2996 (10.7)	18 (11.0)	1214 (11.0)	6 (9.8)	1782 (10.2)	12 (11.8)
Environmental smoking during childhood						
No	16,375 (58.6)	97 (59.5)	6495 (59.0)	37 (60.7)	9880 (58.4)	60 (58.8)
Yes	8599 (30.8)	48 (29.4)	3293 (29.9)	18 (29.5)	5306 (31.3)	30 (33.3)
Missing	2957 (10.6)	18 (11.0)	1214 (11.0)	6 (9.8)	1743 (10.3)	12 (11.8)
Alcohol (g/day)						
Mean	10.7	10.6	15.5	14.7	7.7	8.1
Median	7.2	6.1	11.4	8.6	5.4	4.9
Missing	0	0	0	0	0	0
DM >24 months prior ca/end						
Yes	4522 (16.2)	25 (15.3)	2276 (20.7)	14 (23.0)	2246 (13.3)	11 (10.8)
No	23,409 (83.8)	138 (84.7)	8726 (79.3)	47 (77.0)	14,683 (86.7)	91 (89.2)
DM <24 months prior ca/end						
Yes	657 (2.4)	27 (16.6)	286 (2.6)	13 (21.3)	371 (2.2)	14 (13.7)
No	27,274 (97.6)	136 (83.4)	10,716 (97.4)	48 (78.7)	16,558 (97.8)	88 (86.3)
Height (cm)						
Mean	168.7	168.3	176.4	177.2	163.6	162.9
Median	168.0	168.0	176.0	177.0	164.0	163.0
Missing	41	0	16	0	25	0
Weight (kg)						
Mean	73.4	73.9	81.7	83.2	68.0	68.4
Median	72.0	72.0	81.0	80.0	66.0	66.0
Missing	42	0	16	0	26	0
Body fat (%)						
Mean	26.8	27.1	20.8	19.9	30.8	31.5
Median	27.0	29.0	20.0	19.0	31.0	32.0
Missing	172	1	77	0	95	1
Hip (cm)						
Mean	98.4	99.3	99.3	99.6	97.9	99.1
Median	98.0	98.0	99.0	98.0	97.0	97.5
Missing	59	0	23	0	36	0
Waist (cm)						
Mean	84.1	85.4	93.7	94.7	77.9	79.9
Median	83.0	84.0	93.0	93.0	76.0	78.5
Missing	57	0	23	0	34	0
WHR (cm/cm)						
Mean	0.85	0.86	0.94	0.95	0.79	0.80
Median	0.84	0.84	0.94	0.95	0.79	0.80
Missing	62	0	24	0	38	0
BMI (kg/m^2^)						
Mean	25.7	26.0	26.3	26.4	25.4	25.8
Median	25.3	25.3	26.0	25.7	24.7	25.1
Missing	42	0	16	0	26	0

### Risk of pancreatic cancer in relation with sex, age, smoking, alcohol consumption, and diabetes

Age-adjusted risk of incident pancreatic cancer in relation with sex, smoking, ETS at work, alcohol consumption, and diabetes is shown in Table 2. A significantly higher risk of incident pancreatic cancer was seen among regular smokers (hazard ratio (HR) 2.86, 95% confidence interval (CI) 1.92–4.27) and occasional smokers (HR 2.74, 95% CI 1.40–5.34), compared to never smokers in the entire cohort, as well as among women selectively (HR 2.66, 95% CI 1.64–4.32 and HR 3.35, 95% CI 1.54–7.31, respectively). Among men, only regular smokers were shown to have a significantly increased risk (HR 3.49, 95% CI 1.67–7.29) compared to never smokers. Exposure to ETS at work for more than 20 years was significantly linked to an increased risk of pancreatic cancer in the entire cohort (HR 2.03, 95% CI 1.37–3.02), and among women (HR 2.30, 95% CI 1.40–3.77), but not among men. Pancreatic cancer risk in the entire cohort and among women selectively was significantly linked to the time of exposure to ETS at work. In the group of never smokers, however, there was no increased risk observed regarding ETS at work (data not shown). However, if grouping never and former smokers together, ETS at work for more than 20 years was significantly associated with an increased pancreatic cancer risk in the entire cohort (HR 1.98, 95% CI 1.15–3.41) and among women (HR 2.03, 95% CI 1.02–4.02), but not among men.

**Table 2:** Risk of incident pancreatic cancer in relation with sex, age, smoking, alcohol consumption, and prevalent diabetes

	Entire cohort		Men		Women		*p* for interaction
	*n*	HR	*n*	HR	*n*	HR	
Sex							
Men	11,063	1.00					
Women	17,031	1.08 (0.79–1.48)					
*p*		0.636					
Age							
Years	28,094	1.05 (1.02–1.07)	11,063	1.02 (0.98–1.06)	17,031	1.06 (1.03–1.09)	0.069
*p*		<0.001		0.362		<0.001	
Smoking							
Never	10,642	1.00	3117	1.00	7525	1.00	
Former	9505	1.43 (0.94–2.16)	4781	1.55 (0.74–3.26)	4724	1.46 (0.87–2.45)	0.545
Occasionally	1261	2.74 (1.40–5.34)	532	1.96 (0.54–7.13)	729	3.35 (1.54–7.31)	0.368
Regularly	6674	2.86 (1.92–4.27)	2627	3.49 (1.67–7.29)	4047	2.66 (1.64–4.32)	0.393
*p* trend		<0.001		<0.001		<0.001	
Environmental tobacco smoke at work							
Never	12,225	1.00	3760	1.00	8465	1.00	
For <10 years	3297	1.44 (0.86–2.41)	1093	1.99 (0.83–4.74)	2204	1.23 (0.64–2.34)	0.429
For 10–20 years	3371	1.40 (0.84–2.34)	1259	1.71 (0.72–4.07)	2112	1.25 (0.65–2.34)	0.703
For >20 years	6259	2.03 (1.37–3.02)	3745	1.86 (0.97–3.58)	2514	2.30 (1.40–3.77)	0.256
*p* trend		0.001		0.082		0.002	
Alcohol							
g/day	28,094	1.00 (0.99–1.02)	11,063	1.00 (0.98–1.01)	17,031	1.01 (0.99–1.04)	0.352
*p*		0.638		0.737		0.206	
Diabetes >24 months prior pancreatic cancer or endpoint							
No	23,547	1.00	8773	1.00	14,774	1.00	
Yes	4547	0.86 (0.58–1.36)	2290	1.09 (0.60–1.98)	2257	0.72 (0.38–1.34)	0.366
*p*		0.576		0.784		0.299	

Adjusted for age and sex

High age was significantly associated with an increased risk of pancreatic cancer in the entire cohort (HR 1.05, 95% CI 1.02–1.07) and among women separately (HR 1.06, 95% CI 1.03–1.09), but not among men. Alcohol consumption was not significantly associated with risk of pancreatic cancer, neither in the entire cohort nor in subgroup analysis according to sex. Prevalent diabetes did not correlate significantly with risk of pancreatic cancer.

Risk of pancreatic cancer in relation with sex, age, smoking, ETS at work, alcohol consumption, and diabetes in the fully adjusted model is shown in Table 3, with similar HRs for age (HR 1.06, 95% CI 1.03–1.08 in the entire cohort and HR 1.07, 95% CI 1.05–1.10 among women), occasional smoking (HR 0.74, 95% CI 1.40–1.35 in the entire cohort and HR 3.29, 95% CI 1.50–7.19 among women), and exposure to ETS at work (HR 1.73, 95% CI 1.15–2.58 in the entire cohort and HR 2.01, 95% CI 1.21–3.31 among women).

**Table 3:** Risk of incident pancreatic cancer in relation with sex, age, smoking, alcohol consumption, and prevalent diabetes

	Entire cohort		Men		Women		*p* for interaction
	*n*	HR	*n*	HR	*n*	HR	
Sex							
Men	11,057	1.00					
Women	17,025	1.14 (0.81–1.60)					
*p*		0.454					
Age							
Years	28,079	1.06 (1.03–1.08)	11,054	1.02 (0.99–1.06)	17,022	1.07 (1.05–1.10)	0.045
*p*		<0.001		0.216		<0.001	
Smoking							
Never	10,642	1.00	3117	1.00	7525	1.00	
Former	9505	1.43 (0.94–2.17)	4781	1.58 (0.75–3.33)	4724	1.44 (0.86–2.42)	0.551
Occasionally	1261	2.74 (1.40–5.35)	532	2.00 (0.55–7.31)	729	3.29 (1.50–7.19)	0.367
Regularly	6674	2.87 (1.92–4.28)	2627	3.57 (1.70–7.47)	4047	2.62 (1.61–4.27)	0.397
*p* trend		<0.001		<0.001		<0.001	
Environmental tobacco smoke at work							
Never	12,222	1.00	3759	1.00	8463	1.00	
For <10 years	3296	1.49 (0.89–2.49)	1092	1.95 (0.82–4.66)	2204	1.29 (0.68–2.47)	0.450
For 10–20 years	3370	1.30 (0.78–2.17)	1259	1.53 (0.64–3.66)	2111	1.17 (0.61–2.24)	0.607
For >20 years	6258	1.73 (1.15–2.58)	3744	1.45 (0.74–2.84)	2514	2.01 (1.21–3.31)	0.259
*p* trend		0.011		0.359		0.013	
Alcohol							
g/day	28,079	1.00 (0.99–1.01)	11,054	1.00 (0.98–1.01)	17,022	1.01 (0.99–1.03)	0.469
*p*		0.999		0.528		0.444	
Diabetes >24 months prior pancreatic cancer or endpoint							
No	23,537	1.00	8768	1.00	14,769	1.00	
Yes	4545	0.88 (0.57–1.34)	2289	1.07 (0.59–1.95)	2256	0.72 (0.38–1.34)	0.358
*p*		0.545		0.819		0.300	

Adjusted for sex, age, smoking, alcohol consumption, and diabetes

In order to assess potential dose-dependent effects, we also examined the risk of pancreatic cancer according to tertiles of alcohol consumption in both the age-adjusted and fully adjusted model, but found no significant associations (data not shown).

In the fully adjusted model (Table 3), there was a significant interaction between age and female sex (*p* for interaction = 0.045) in relation with pancreatic cancer risk. There was no significant interaction with any of the other factors and sex, neither in the age-adjusted nor in the fully adjusted model.

ETS during childhood and at home were not significantly associated with pancreatic cancer risk, neither in the entire cohort nor in sex-stratified analysis (data not shown).

### Risk of pancreatic cancer in relation with anthropometric factors

Risk of incident pancreatic cancer in relation with anthropometric factors is shown in Table 4.

**Table 4:** Risk of incident pancreatic cancer in relation with anthropometric factors

	Entire cohort		Men		Women	
	*n*	HR	*n*	HR	*n*	HR
Height tertiles						
1	9859	1.00	3711	1.00	6138	1.00
2	9080	0.75 (0.50–1.12)	3838	0.98 (0.53–1.83)	5439	1.04 (0.66–1.65)
3	9102	0.76 (0.44–1.32)	3492	1.17 (0.63–2.18)	5423	1.00 (0.61–1.64)
*p* trend		0.246		0.630		0.986
Weight tertiles						
1	9475	1.00	3819	1.00	5849	1.00
2	9337	1.09 (0.74–1.61)	3504	1.27 (0.69–2.34)	5652	1.15 (0.72–1.82)
3	9228	1.22 (0.79–1.89)	3718	1.11 (0.58–2.13)	5498	1.00 (0.61–1.65)
*p* trend		0.379		0.731		0.969
BMI tertiles						
1	8430	1.00	3463	1.00	6165	1.00
2	9166	1.29 (0.86–1.93)	4099	1.12 (0.61–2.07)	5178	1.28 (0.78–2.09)
3	10,444	1.34 (0.89–2.02)	3479	1.06 (0.54–2.09)	5656	1.34 (0.82–2.19)
*p* trend		0.167		0.852		0.249
Hip tertiles						
1	9395	1.00	3973	1.00	5880	1.00
2	9809	1.50 (1.02–2.20)	3371	0.89 (0.48–1.67)	5377	1.54 (0.94–2.52)
3	8819	1.31 (0.86–1.99)	3690	1.00 (0.54–1.85)	5732	1.35 (0.81–2.27)
*p* trend		0.213		0.980		0.270
Waist tertiles						
1	9144	1.00	3872	1.00	5884	1.00
2	9458	1.47 (0.98–2.21)	3639	1.10 (0.61–2.00)	5509	1.47 (0.90–2.40)
3	9423	1.58 (0.97–2.57)	3523	0.90 (0.47–1.75)	5598	1.35 (0.81–2.26)
*p* trend		0.063		0.792		0.264
WHR tertiles						
1	9294	1.00	3531	1.00	5204	1.00
2	9199	1.34 (0.90–1.99)	3842	1.15 (0.61–2.19)	6458	1.23 (0.75–2.02)
3	9527	2.36 (1.28–4.35)	3660	1.35 (0.71–2.57)	5325	1.35 (0.81–2.26)
*p* trend		0.009		0.360		0.256
BF% tertiles						
1	9415	1.00	3748	1.00	5352	1.00
2	9055	1.12 (0.69–1.83)	3697	0.88 (0.48–1.59)	6476	1.65 (1.00–2.72)
3	9439	1.51 (0.87–2.62)	3535	0.79 (0.42–1.50)	5101	1.33 (0.76–2.33)
*p* trend		0.108		0.472		0.353

Adjusted for sex, age, smoking, alcohol consumption, and diabetes

In the entire cohort, the risk of incident pancreatic cancer was significantly higher for individuals with a high WHR (HR 2.36, 95% CI, 1.28–4.35 for tertile 3, compared with the lowest tertile, *p* for trend = 0.009). This significant difference was not observed in subgroup analysis according to sex. There was a borderline significant association of high waist circumference with pancreatic cancer risk in the entire cohort (*p* for trend = 0.063), but not in subgroup analysis according to sex.

Beyond these associations, none of the other examined factors were found to be significantly associated with an increased risk of incident pancreatic cancer.

Inclusion of WHR in the fully adjusted model in Table 3 did not alter the results except for a significantly increased risk of pancreatic cancer in women compared to men (HR 2.02, 95% CI, 1.17–3.47) and a borderline significant interaction between female sex and age in relation with risk (*p* for interaction = 0.060).

## Discussion

In this study, we have examined the associations of anthropometric factors, and two other major lifestyle-related risk factors, smoking and alcohol consumption, with risk of pancreatic cancer, with particular reference to potential differences between sexes, in the Malmö Diet and Cancer study.

As expected, the results confirm smoking as an important risk factor for incident pancreatic cancer, overall and in both sexes. This association was particularly evident in regular smokers, but even occasional smoking was shown to be significantly associated with risk of pancreatic cancer in women, but not in men. An additional difference between sexes was observed in that exposure to ETS at work for more than 20 years was found to be significantly associated with an increased risk of pancreatic cancer in women, but not in men. This association remained significant in the group of never and former smokers; however, in contrast to the results in the study on the EPIC cohort, no significant correlations to risk were seen in the group of only never smokers regarding ETS [23]. The finding of a possibly stronger correlation between smoking and risk of pancreatic cancer in women is in line with the results of several previous studies [10, 15, 23, 38]. The interaction between smoking and sex was tested in three of these studies, whereby two found no significant interaction [23, 38] and one a significant interaction between sex and duration of smoking [10]. Whether women are more susceptible to the carcinogenic effects of smoking than men has been investigated previously, in particular in studies on lung cancer [39–42]. Collectively, these studies indicate an increased vulnerability to tobacco carcinogens among women, but whether this is also the case in pancreatic cancer has not yet been thoroughly investigated. However, the obvious link between smoking and pancreatic cancer risk, and the herein observed potential sex differences, notwithstanding the non-significant interaction between sex and smoking or exposure to ETS at work, emphasizes the importance to consider potential sex differences in epidemiological studies. Moreover, despite the still lower reported percentage of smokers among women than among men, a plausible explanation for the current equal incidence rate of pancreatic cancer between the sexes may well be the rising female-to-male smoking prevalence ratios in high-income countries, where pancreatic cancer is more common than in low-income countries [43, 44].

It is noteworthy that former smokers were not shown to have an increased risk of incident pancreatic cancer in the present study. One could ponder that regular smoking would be a potential risk factor not only when ongoing but also for a period of time after quitting. A previous study on the EPIC cohort found that both regular smokers and former smokers, who had smoked for more than 25 years or quit less than 5 years prior to study entry, had an increased risk of incident pancreatic cancer [23]. Those who quit more than 5 years before study entry did however not have a significantly increased risk, and there was no significant heterogeneity between sexes regarding risk for current smokers compared to never smokers. Among never smokers, pancreatic cancer risk was also shown to be significantly increased for those who had been exposed to ETS during childhood and at work or at home in their adult life, compared to those never exposed. Those only exposed to ETS at home or work in their adult life had a borderline significantly increased pancreatic cancer risk, but those only exposed in childhood had no significantly increased risk [23].

In the present study, alcohol consumption did not differ significantly between cases and non-cases and was not found to be significantly associated with pancreatic cancer risk, neither in the entire cohort nor in sex-stratified analysis. High alcohol consumption is a well-known risk factor for pancreatitis [31, 32], and pancreatitis has, in some studies, been shown to be a risk factor for incident pancreatic cancer [7, 16, 17]. Along this line, alcohol consumption can be assumed to influence pancreatic cancer risk, but the results from several previous studies in this regard are inconclusive [24–30]. Potential sex differences are even less investigated, but, according to existing data, the correlation appears to be stronger among men [25–27].

Prevalent diabetes has in many studies been highlighted as a risk factor for pancreatic cancer [13–15]. According to the results of the present study, however, only new-onset diabetes was found to be significantly more common among cases compared with non-cases. This finding is in line with the expected, as the majority of patients with pancreatic cancer are known to develop diabetes at some point due to their disease [2, 4, 8, 45]. Along this line, it will be of interest to further interrogate the associations of various factors associated with insulin resistance and pancreatic cancer risk in the herein investigated cohort.

The observation that age at baseline was higher among cases compared with the remaining cohort is expected, since the incidence of pancreatic cancer in Sweden in 2014 peaked at the age of 70–74 years, and the median age at baseline for the entire cohort of this study was 57.8 years, with the oldest individual being 74 years. Hence, the individuals entering the study at a higher age than the median were closer to their age of peak incidence. Of note, this significant difference was only seen in the entire cohort, and among women, with a significant interaction between female sex and age in the fully adjusted model.

Another purpose of this study was to investigate the relationship between pre-diagnostic anthropometry and risk of pancreatic cancer, overall and according to sex. Apart from the finding of a high WHR being significantly associated with pancreatic cancer risk in the entire cohort, but not in sex-stratified analysis, none of the other investigated anthropometric factors was significantly associated with pancreatic cancer risk, neither in the entire cohort nor in sex-stratified analyses. According to the existing literature, BMI has been proposed as the anthropometric factor with the strongest association to risk of pancreatic cancer, but yet there is no definite consensus [5–7, 15, 16, 18, 19]. Several of the previous studies reporting significant correlations between overweight/obesity and an increased risk of pancreatic cancer have shown this to apply to both sexes [5, 19]; however, in the pooled study performed by Genkinger et al., modest differences between sexes were observed regarding the association of BMI with pancreatic cancer risk [19]. In a previous study within the EPIC cohort, including incident cases in the MDCS up until April 2004, a higher WHR as well as a larger waist circumference was observed to significantly increase the risk of pancreatic cancer, while there were no significant associations between BMI and risk of pancreatic cancer [20]. In light of the herein presented results, it would be of interest to re-examine the associations of pre-diagnostic anthropometry with pancreatic cancer risk in the EPIC cohort, with a more recent follow-up on incident cases.

Of note, the number of female participants in the MDCS is higher than the number of male participants, which resulted in a higher number of female than male pancreatic cancer cases. However, the incidence rate of pancreatic cancer did not differ significantly between men and women, which confirms the contemporary equal incidence rate of pancreatic cancer between the sexes [1, 3].

## Conclusions

In this study, regular smoking was confirmed to be a strong risk factor for pancreatic cancer in both sexes, but the relationship between occasional smoking and long-term environmental exposure with pancreatic cancer risk was only observed in women. Despite the lack of a significant interaction with sex, these findings suggest that the carcinogenic effects of smoking may be more hazardous in women and emphasize the importance of taking potential sex differences into consideration in epidemiological studies and prevention efforts. Furthermore, the results from this study demonstrate that a high waist-hip ratio was the only pre-diagnostic anthropometric factor being significantly associated with pancreatic cancer risk, with no difference between sexes.

## Abbreviations

BMI: Body mass index
CI: Confidence interval
CP: Chronic pancreatitis
DM: Diabetes mellitus
EPIC: European Prospective Investigation into Cancer and Nutrition
ETS: Environmental tobacco smoke
HR: Hazard ratio
MDCS: Malmö Diet and Cancer Study
OS: Overall survival
RAP: Recurrent acute pancreatitis
WHR: Waist-hip ratio
